# Vascular Structure and Function in Children and Adolescents: What Impact Do Physical Activity, Health-Related Physical Fitness, and Exercise Have?

**DOI:** 10.3389/fped.2020.00103

**Published:** 2020-03-19

**Authors:** Lisa Baumgartner, Heidi Weberruß, Renate Oberhoffer-Fritz, Thorsten Schulz

**Affiliations:** Institute of Preventive Pediatrics, TUM Department of Sport and Health Sciences, Technical University of Munich, Munich, Germany

**Keywords:** vascular structure, vascular function and stiffness, health-related physical fitness, physical activity, exercise, children, adolescents

## Abstract

A physically active lifestyle can prevent cardiovascular disease. Exercise intervention studies in children and adolescents that aim to increase physical activity have resulted in reduced vascular wall thickening and improve cardiovascular function. Here we review the literature that explores the correlations between physical activity, health-related physical fitness, and exercise interventions with various measures of vascular structure and function in children and adolescents. While several of these studies identified improvements in vascular structure in response to physical activity, these associations were limited to studies that relied on questionnaires. Of concern, these findings were not replicated in studies featuring quantitative assessment of physical activity with accelerometers. Half of the studies reviewed reported improved vascular function with increased physical activity, with the type of vascular measurement and the way physical activity was assessed having an influence on the reported relationships. Similary, most of the studies identified in the literature report a beneficial association of health-related physical fitness with vascular structure and function. Overall, it was difficult to compare the results of these studies to one another as different methodologies were used to measure both, health-related physical fitness and vascular function. Likewise, exercise interventions may reduce both arterial wall thickness and increased vascular stiffness in pediatric populations at risk, but the impact clearly depends on the duration of the intervention and varies depending on the target groups. We identified only one study that examined vascular structure and function in young athletes, a group of particular interest with respect to understanding of cardiovascular adaptation to exercise. In conclusion, future studies will be needed that address the use of wall:diameter or wall:lumen-ratio as part of the evaluation of arterial wall thickness. Furthermore, it will be critical to introduce specific and quantitative measurements of physical activity, as intensity and duration of participation likely influence the effectiveness of exercise interventions.

## Introduction

Cardiovascular disease (CVD) is currently the leading cause of death worldwide; in 2030, CVD may be the major underlying factor in 22.2 million deaths per year (1). To prevent CVD, the American Heart Association recommends adopting a physically active lifestyle, healthy diet and the avoidance of tobacco use (2). Cardiovascular (CV) parameters, including intima-media thickness (IMT) and pulse-wave-velocity (PWV) as subclinical risk maker for CVD, should be monitored at an early age to identify and follow children and adolecents who are at higher risk for CV events (3).

Carotid IMT (cIMT) is an important surrogate marker of subclinical atherosclerosis. It can be assessed using ultrasound at a young age and long before atherosclerotic symptoms occur. Although the incidence of carotid plaques is low in this age group, cIMT may be elevated in children and adolescents with obesity (4, 5), hypertension (6), diabetes (7), cancer (8), or some types of congenital heart disease (9).

The Association for European Paediatric Cardiology Working Group on Cardiovascular Prevention recommends measuring cIMT in pediatric populations using high-resolution ultrasound in two different angels after the carotid bulb of the left and right common carotid artery with a slightly stretched neck and head turned 45° to the opposite side (10). IMT can also be measured at the abdominal aorta as well as at the femoral artery. Of note, arterial diameters can also be measured; these values will permit one to calculate IMT:diameter-ratio (alternatively wall:lumen-ratio) which expresses the relation of wall thickness (IMT) to vascular diameter or lumen, respectively. These ratios are helpful for determining the full impact of physical activity (PA) and other interventions on vascular structure and function.

Vascular compliance is a measure of arterial function that describes the physical adaptation to the blood volume ejected by the left ventricle (LV) and reflects the ability to maintain constant blood flow (11). Vascular stiffness is the reciprocal of vascular compliance and is defined by different parameters such as elastic modulus or pulse wave velocity (PWV). Vascular stiffness can be measured with ultrasound, oscillometric devices, photoplethysmography, and/or applanation tonometry applied to the aorta, femoral, brachial or carotid arteries. Parameters measured with this devices include compliance, distensibility, stiffness index, reflection index, elastic modulus, augmentation index (AI), and PWV. Pediatric populations with elevated cardiovascular risk can present reduced compliance or increased stiffness, for example children with hypertension (12), obesity (12, 13), hypercholesterolemia (14), metabolic syndrome (15), diabetes (16), and congenital heart disease (17).

PA and health-related physical fitness (HRPF) are both associated with a lower risk of CVD in adulthood (18, 19). The results of several recent studies suggest a critical role for PA and HRPF in promoting healthy vascular structure and function in children and adolescents (20, 21). Interventions that include endurance or resistance exercises have also been introduced in an attempt to improve impaired vascular structure and function in pediatric populations at risk (22, 23).

This review provides an overview of the relationship between PA and HRPF with vascular structure and function in children and adolescents. Exercise is a planned and structured PA to improve activity level and HRPF. Therefore, this review further discusses the effect of exercise on vascular structure and function in intervention studies and young athletes. We searched in several databases for articles published between 2005 and June 2019 using the keywords “intima-media thickness,” “arterial structure,” “arterial diameter,” “vascular function,” “arterial stiffness,” “exercise,” “physical activity,” “physical fitness,” “cardiorespiratory fitness,” “athlete,” “child^*^,” or “adolesc^*^.” We did not include a discussion of biochemical parameters of endothelial function in the review.

## Physical Activity

PA is any movement of the body that is produced by skeletal muscles and that requires energy expenditure (24). Regular PA has a cardioprotective effect on health not only in youth but also later in life. Therefore, children should adopt a physically active lifestyle at an early age and maintain it throughout life. The World Health Organization recommends that children and adolescents undertake moderate-to-vigorous physical activity (MVPA) for at least 60 min per day (Table 1) (24). MVPA is commonly assessed as self-reporting using specific questionnaires. While this method is simple, the data that result may be inaccurate due to recall bias (28). Accelerometer and related wearable instruments permit collection of more objective data, as these devices do not depent on self-assessment. PA measurements vary by design and as per the individual goal for the research program (29).

**Table 1:** Description of MET and activity intensity levels.

MET	1 MET = consumption of 3.5 ml O_2_ per kilogram body mass per minute at rest (25)
SED	≤1.5 METs during sitting, reclining or lying position (26)
LPA	<3.0 METs, e.g., walking slowly, washing dishes or playing darts (27)
MPA	3.0–6.0 METs, e.g., washing windows, golf, recreational badminton (27)
VPA	>6.0 METs, e.g., jogging, cross-country skiing, soccer (27)
MVPA	≥3 METs

*MET, metabolic equivalent; SED, sedentary behavior; LPA, light physical activity; MPA, moderate physical activity; VPA, vigorous physical activity; MVPA, moderate-to-vigorous physical activity*.

### Vascular Structure

We identified six published studies that explored correlations between PA and vascular structure (Table 2); four of these reports are included in the literature review published in 2016 by Cayres et al. (41). Three of the six studies used accelerometers to measure PA (20, 33, 37) and three relied on questionnaires (31, 35, 36).

**Table 2:** Overview of studies that evaluated associations of physical activity and vascular structure and function.

**References**	***N***	**Age**	**Measurement of PA**	**Vascular measurement**	**Parameter of vascular structure**	**Parameter of vascular function**	**Results**	**Statistics**
Haapala et al. (30)	136	6–8	Combined heart rate and accelerometer	Photoplethysmography		Stiffness index	↓stiffness index with ↑MPA	β = −0.273, 95% CI−0.448 to−0.097, *p* = 0.003
							↓stiffness index with ↑VPA	β = −0.254, 95% CI−428 to−0.080, *p* = 0.005
							↓stiffness index with cumulative time spent in PA > 3 METs	β = −0.279, 95% CI−0.453 to−0.106, *p* = 0.002
							↓stiffness index with cumulative time spent in PA > 4 METs	β = −0.341, 95% CI−0.515 to−0.167, *p* < 0.001
							↓stiffness index with cumulative time spent in PA > 5 METs	β = −0.349, 95% CI−0.524 to−0.174, *p* < 0.001
							↓stiffness index with cumulative time spent in PA > 6 METs	β = −0.312, 95% CI−0.220 to−0.064, *p* < 0.001
							↓stiffness index with cumulative time spent in PA > 7 METs	β = −0.254, 95% CI−0.428 to−0.080, *p* = 0.005
Idris et al. (31)	595 237	5 8	Questionnaire	Ultrasound	cIMT	Carotid distensibility Carotid elastic modulus	No association of total MET and vascular parameters	-
							↓cIMT with ↑sport time-weighted MET at 5yrs	−3.20 mm/SD, 95% CI−6.34 to−0.22, *p* = 0.04
							No association of organized sport time-weightes MET at 5yrs	-
							No association of total, sport and organized sport time-weights MET at 8yrs	-
Kochli et al. (32)	1171	6–8	Questionnaire	Oscillometric device		Aortic PWV	No association of VPA, indoort and outdoor activity and aortic PWV	-
Melo et al. (33)	265	11–13	Accelerometer	Ultrasound	cIMT		No association of SED and MVPA with cIMT	-
Nettlefold et al. (34)	102	8–11	Accelerometer	Applanation tonometry		Compliance of small and large arteries	No association of PA, MPVA, SED, LPA, MPA and VPA and compliance of large arteries	-
							↑compliance of small arteries with ↑LPA	*p* = 0.003
							↑compliance of small arteries with ↑MPA	*p* = 0.036
							↑compliance of small arteries with ↑MVPA	*p* = 0.043
Pahkala et al. (35)	553 531 494	13 15 17	Questionnaire	Ultrasound	aIMT		↓aIMT with ↑LTPA	β ± SD = −0.00034 ± 0.00014, *p* = 0.011
							↓progression of aIMT with a moderate increase in LTPA between 13 and 17	*p* = 0.047
Pahkala et al. (36)	449-467	17	Questionnaire	Ultrasound	aIMT, cIMT		No association between aIMT and LTPA	-
							No association between cIMT and LTPA	-
							↓aIMT in high LTPA compared to low LTPA concerning fitness level	*p* = 0.019
Ried-Larsen et al. (37)	397	15.6 ± 0.4	Accelerometer	Ultrasound	cIMT	Carotid compliance Carotid distensibility Carotid YEM Stiffness index	No association between MVPA, VPA and vascular parameters	-
							Lower carotid YEM in the highest quartile of MVPA in boys	*p* < 0.05
							Lower stiffness index in the highest quartile of MVPA in boys	*p* < 0.05
Ried-Larsen et al. (20)	254	8–10	Accelerometer	Ultrasound	cIMT	Carotid compliance Carotid distensibility Carotid YEM Stiffness index	No association between MVPA or VPA and vascular parameters	-
Ried-Larsen et al. (38)	375	15.7 ± 0.4	Questionnaire	Ultrasound		Carotid compliance Carotid distensibility Carotid YEM	↑carotid compliance in boys who practice bicycling every day	β = 0.44, 95% CI 0.08 to 0.81, *p* = 0.02
							↑carotid distensibility in boys who practice bicycling every day	β = 0.40, 95% CI 0.02 to 0.77, *p* = 0.04
							↓carotid YEM in boys who practice bicycling every day	β = −0.50, 95% CI−0.86 to−0.13, *p* = 0.01
							↑carotid compliance in boys who use bike for traveling to school	β = 0.59, 95% CI 0.08 to 1.01, *p* = 0.02
							↓carotid YEM in boys who use bike for traveling to school	β = −0.54, 05% CI−1.07 to−0.02, *p* = 0.045
							No associations in girls	-
Veijalainen et al. (39)	160	6–8	Questionnaire	Photoplethysmography		Stiffness index reflection index	↓stiffness index with ↑unstructured PA	β = - 0.162, *p* = 0.042
							No association of stiffness index and total PA and recess PA	-
							No association between reflection index and unstructured, total and recess PA	-
Walker et al. (40)	485	12–14	Questionnaire	Applanation tonometry		Carotid-femoral PWV Aortic AI	No association of carotid-femoral PWV and aortic AI with PA	-

*PA, physical activity; Cimt, carotid intima-media thickness; aIMT, aortic intima-media thickness; PWV, pulse wave velocity; YEM, young's elastic modulus; AI, augmentation index; MPA, moderate physical activity; VPA, vigorous physical activity; MVPA, moderate to vigorous physical activity; MET, metabolic equivalent; SED, sedentary behavior; LPA, light physical activity; LTPA, leisure time physical activity*.

Among the results of the questionnaire-based studies, Idris et al. (31) found no relationship between time-weighted metabolic equivalents (MET, Table 1) and cIMT but identified lower cIMT in association with higher values of time-weighted sports-related MET among the 5-year old participants. Likewise, Pahkala et al. (35) found that leisure-time physical activity (LTPA) had a beneficial impact on aortic IMT (aIMT); specifically, a moderate increase in LTPA among sedentary 13 and 15 year olds was associated with a significant decreased progression of aIMT. In a similar study, Pahkala et al. (36) reported no direct association between LTPA and cIMT or aIMT, although 17-year olds with high levels of LTPA overall experienced lower levels of aIMT compared to those with low LPTA in relation to their fitness level.

By contrast, among the studies that made use of accelerometers, Ried-Larsen et al. (20, 37) reported no correlation between MVPA or vigorous PA (VPA) and cIMT in a study of 8- to-10-year old Danish children; analogous results were obtained from adolescents with a mean age of 15.6 ± 0.4 years. Similary, Melo et al. (33) found that sedentary behavior (SED) and MVPA were not definitively related to cIMT in 11- to-13-year-old Portuguese children and adolescents.

In summary, we conclude that studies that used questionnaires identified correlations between PA and healthy vascular structure, but no such relationship emerged from studies that employed accelerometers. As such, it is clear that associations between PA and vascular structure may depend all or in part on the method used to assess activity. Nonetheless, three out of six studies reported beneficial associations between PA and cIMT or aIMT.

### Vascular Function

We identified eight studies that examined the relationships between PA and vascular function; five of these studies used questionnaires (31, 32, 38–40) and three (20, 30, 34) used accelerometers to assess PA in children and adolescents.

Moderate PA (MPA), VPA and cumulative time spent in PA, specifically data documenting individuals achieving above 3–7 METs, were inversely associated with stiffness index, measured at the finger tip by pulse contour analysis. Of note, children participating in PA who achieved 3 METs/day presented with lower stiffness index values (30). Nettlefold et al. (34) found no association between PA and compliance of the large arteries, but did report higher compliance of small arteries with increased time spent in light PA (LPA), MPA, or MVPA per day. Likewise, Ried-Larsen et al. (38) found that boys who ride bicycles every day have lower young's elastic modulus, higher distensibility, and higher arterial compliance compared to boys who ride fewer than three times per week. Likewise, boys who traveled to school by bicycle had higher compliance and lower young's elastic modulus compared to those who use passive transportation (38).

In other studies, more time spent in unstructured PA was related to lower stiffness index in children between 6 and 8 years of age (39). However, Ried-Larsen et al. (37) did not identify a direct association between time spent in MVPA and carotid compliance, distensibility, young's elastic modulus, and stiffness index; there was also no statistical relationship between VPA and compliance, distensibility, young's elastic modulus, and stiffness index. However, boys classified in the highest quartile of MVPA had significantly lower young's elastic modulus and stiffness index compared to boys in the lowest quartile.

By contrast, carotid distensibility and carotid elastic modulus were associated with neither total time-weighted MET, sports time-weighted MET nor organized sport time-weighted MET in children (31). Furthermore, parameters relating to vascular stiffness were not associated with time spent in MVPA in a daily basis and VPA in children and adolescents (20). VPA (categorized in tertiles) had no impact on carotid-femoral PWV nor on aortic AI (40). Kochli et al. (32) also found no association between aortic PWV and VPA, indoor or outdoor activity.

In conclusion, of the eight studies that explored the association between PA and vascular stiffness, four reported improved vascular function with increased PA; two of these studies reported accelerometer findings and two used questionnaires. Of importance, none of the studies identified any unfavorable associations between PA and vascular stiffness. The two studies which used photoplethysmography reported significant lower stiffness indices in association with higher levels of PA (30, 39). The four studies reported ultrasound measurements of vascular parameters and the study which used PA questionnaires all report an inverse correlation between PA and vascular stiffness (38); this result was not reported in the two studies which used accelerometers for measuring PA (20, 37). Of the studies which used applanation tonometry to measure vascular stiffness, only Nettlefold et al. (34) reported better vascular elasticity with increasing PA; of note, this study featured accelerometer findings to report PA in contrast to the report from Walker et al. (40) which used a questionnaire. Hence, the type of vascular measurement employed and the means by which PA was assessed may have an influence on the relationships reported. Of note, the number of study participants in each study may also have an influence on the results obtained.

## Health-Related Physical Fitness

HRPF includes cardiorespiratory fitness (CRF), muscular endurance, muscular strength, flexibility, and body composition and is an important indicator of health and well-being (42). The following studies focus on CRF or strength and include healthy children and adolescents only and exclude those with chronic conditions including overweight, obesity or diabetes.

### Vascular Structure

The majority of the studies included in this review used ergometers to measure HRPF (Table 3) and include results from single trails that include a 20 m shuttle run, handgrip strength test, curl-ups, and push-ups.

**Table 3:** Overview of studies that evaluated associations of health-related physical fitness and vascular structure and function.

**References**	***N***	**Age**	**Measurement of HRPF**	**Vascular measurement**	**Parameter of vascular structure**	**Parameter of vascular function**	**Results**	**Statistics**
Agbaje et al. (43)	329	8–11	Ergometer test	Photoplethysmography		Stiffness index	↑reflection index with ↑CRF in boys	β = 0.377, *p* = 0.001
							↑reflection index with ↑CRF in girls	β = 0.337, *p* = 0.02
							no association between stiffness index and CRF	
Farr et al. (44)	96	9–10	Ergometer test	Applanation tonometry		peripheral non-transformed index carotid-ankle PWV carotid-radial PWV	no linear association between vascular parameters and CRF	-
							↑carotid-ankle PWV in higher CRF group compared to lower CRF group	*p* < 0.05
							no differences in periperhal non-transformed index and carotid-radial PWV betweeh higher and lower CRF group	-
Kochli et al. (32)	1171	6–8	20 m shuttle run	Oscillometric device		Aortic PWV	↓aortic PWV with ↑CRF	β = −0.024, 95% CI−0.035 to−0.012, *p* < 0.001
Melo et al. (33)	265	11–13	Ergometer test	Ultrasound	cIMT		↓cIMT with ↑CRF	β = −0.13, *p* = 0.04
Melo et al. (45)	336	11–12	Hand grip	Ultrasound	cIMT carotid artery diameter		↓cIMT in middle and high strength group compared to low strength group	*p* < 0.05
							no differences in carotid artery diameter between strength groups	
Melo et al. (46)	413	11–12	Ergometer test	Ultrasound	cIMT		OR of 2.8 for unfit children having increased cIMT	95% CI 1.40 - 5.53
Meyer et al. (47)	646	13.9 ± 2.1	6 min run	Oscillometric device		Aortic PWV Aortic AI@75	↑aortic PWV with ↑CRF	β = 0.173, *p* < 0.001
							↓aortic AI@75 with ↑CRF	β = −0.106, *p* = 0.025
Pahkala et al. (36)	449–467	17	Ergometer test	Ultrasound	aIMT, cIMT	Aortic distensibility Carotid distensibility Aortic young's elastic modulus Carotid young's elastic modulus	↓aIMT with ↑CRF	β = −0.0029 ± 0.0013, *p* = 0.031
							no association between cIMT and CRF	-
							↓aortic young's elastic modulus with ↑CRF	β = −0.012 ± 0.0053, *p* = 0.025
							no association with distensibility and carotid young's elastic modulus	-
Reed et al. (48)	99	9–11	20 m shuttle run	Applanation tonometry		Compliance of large and small arteries	↑compliance of large arteries and CRF	not available
							↑compliance of small arteries and CRF	not available
							↑compliance of large arteries in quartile 4 compared to quartile 1 and 2	*p* < 0.05
							↑compliance of small arteries in quartile 4 compared to quartile 2	*p* < 0.05
Ried-Larsen et al. (37)	397	15.6 ± 0.4	Ergometer test	Ultrasound	cIMT	Carotid compliance Carotid distensibility Carotid young's elastic modulus Stiffness index	no association between cIMT and carotid compliance and CRF	-
							↓carotid young's elastic modulus with ↑CRF	β = −16.38, 95% CI−27.16 to−5.60, *p* = 0.003
							↑carotid distensibility with ↑CRF	β = 0.24, 95% CI 0.01 to 0.47, *p* = 0.037
							↓stiffness index with ↑CRF in boys	β = −0.28, 95% CI−0.55 to−0.01, *p* = 0.049
							↓carotid young's elastic modulus in quartiles 2, 3 and 4 comparted to quartile 1	*p* < 0.05
							↓stiffness index in quartiles 3 and 4 compared to quartile 1	*p* < 0.05
							higher carotid distensibility in quartiles 3 and 4 compared to quartile 1	*p* < 0.05
Sakuragi et al. (49)	573	10.1 ± 0.3	20 m shuttle run	Applanation tonometry		Carotid-femoral PWV	↓carotid-femoral PWV with ↑CRF	β = −0.047, 95% CI−0.07 to−0.024, *p* < 0.001
Veijalainen et al. (39)	160	6–8	Ergometer test	Photoplethysmography		Stiffness index Reflection index	↓stiffness index with ↑CRF	β = −0.246, *p* = 0.006
							no association between reflection and CRF	-
Weberruss et al. (21)	697	7–17	20 m shuttle run Curl-ups Push-ups	Ultrasound	cIMT	Carotid elastic modulus Carotid compliance Stiffness index β PWV β	↑cIMT with ↑CRF	β = <0.001 ± 0, *p* < 0.001
							↑carotid compliance with ↑CRF	β = 0.004 ± 0.001, *p* < 0.001
							↓stiffness index β with ↑CRF	β = −0.01 ± 0, *p* < 0.001
							↓PWV β with ↑CRF	β = −0.01 ± 0, *p* < 0.001
							↓carotid elastic modulus with ↑CRF	β = −0.12 ± 0.03, *p* < 0.001
							↑carotid elastic modulus in low fit group	*p* = 0.009
							↑stiffness index β in low fit group	*p* = 0.03
							↑ PWV β in low fit group	*p* = 0.005
							no associations with curl-ups and push-ups	-

*cIMT, carotid intima-media thickness; aIMT, aortic intima-media thickness; PWV, pulse wave velocity; CRF, cardiorespiratory fitness; AI, augmentation index*.

Among Portuguese children between the ages of 11 and 13 years, cIMT was inversely associated with CRF independent of age, sex, maturity, SED, and MVPA (33) and values of maximum oxygen uptake revealed an inverse correlation with aIMT (36). Furthermore, participants identified as “low-fit” at the age of 11 experienced accelerated progression of aIMT between ages of 11 and 17 years (36).

By contrast, in a study involving 697 children and adolescents between 7 and 17 years, Weberruss et al. (21) found a significant positive correlation between cIMT and CRF. CRF was measured with a 20m shuttle run, which means that the participant runs between two lines (20m distance) with a given speed. The speed is stepwise increased and the time will be stopped if the participant does not touch the line in the given speed. In contrast, Pahkala et al. (36) observed no associations between these parameters; diminished CRF was not associated with increased risk of having cIMT. No association of a maximal load test on a cycle ergometer and cIMT was reported in Danish adolescents (37) but Melo et al. (46) reported an OR of 2.8 in unfit children for having a cIMT ≥ 75th percentile.

Muscular strength has also been evaluated as a parameter contributing to HRPF. Among 11- to 12-year old Portuguese children, carotid artery diameter did not differ between children with low, middle or high muscle strength index; but children with middle to high muscle strength index as measured using a handgrip test showed lower values of cIMT compared to those measured in children with low muscular strength (45). By contrast, muscular strength determined in a series of German children and adolescents (push-ups and curl-ups) was not associated with CRF (21).

Taken together, the literature reveals a significant relationship between HRPF and vascular structure in population-based studies that include children and adolescents; in general, higher performance in HRPF tasks corresponds to lower levels of IMT. One study did reveal an inverse correlation between CRF and aIMT but not with cIMT. The authors hypothesize that structural alterations such as IMT may begin in the aorta and progress in the carotid artery later in life (36, 50).

Interestingly, the association between HRPF and vascular structure has occurred primarily in children and adolescents with increased cardiovascular risk; by contrast, the participants in most published studies are their healthy peers. For example, Ried-Larsen et al. (37) enrolled only healthy participants and excluded those who were chronically ill; this may explain the absence of associations observed between cIMT and CRF in this study. Arteries of fit children and adolescents may undergo adaptation to exercise, a process which may have an impact on smooth muscle cells in the vascular media; this adaptation may explain the positive relationship of cIMT to CRF observed by Weberruss et al. (21). Nevertheless, at this time there is no clear association between HRPF (evaluated as muscular strength) and vascular structure; further investigation is warranted.

### Vascular Function

We identified ten studies which explored the association between HRPF and arterial function in children and adolescents; five studies featured a (maximal) ergometer test, one included a 6-min run, three studies included the 20 m shuttle run, and one study explored responses to a combination of the 20 m shuttle run, curl-ups and push-ups. The studies applied numerous methods to measure vascular stiffness (ultrasound, oscillometric device, photoplethysmography, applanation tonometry) and included a wide range of stiffness parameters.

In the first group of studies, CRF was inversely and therefore beneficially associated with aortic PWV but the association became non-significant when the model was further adjusted for blood pressure (32). Carotid PWV (21) and carotid-femoral PWV (49) were both inversely related to CRF adjusted for blood pressure. Farr et al. (44) reported no direct correlation between CRF and carotid-ankle PWV, carotid-radial PWV or peripheral non-transformed index but when devided into a higher and lower group of CRF, subjects in the higher group of CRF had higher carotid-ankle-PWV compared to the lower group. By contrast, another study reported increased aortic PWV in association with higher CRF in children and adolescents between the ages of 11 and 17 years (47). Three of four studies observed a significant favorable association between CRF and stiffness index (21, 37, 39, 43).

Two studies used non-invasive photoplethysmography and assessed reflection index as parameter of vascular function; one identified no correlations between the reflection index and CRF (39), one reported a positive association between reflection index and CRF in boys and girls (43). Pahkala et al. (36) reported an inverse correlation between CRF and young's elastic modulus for the aorta but not for the carotid artery among a group of 17-years olds. Furthermore, Weberruss et al. (21) documented a favorable association between CRF and carotid elastic modulus while Ried-Larsen et al. (37) reported a significant inverse correlation between CRF and carotid elastic modulus.

By contrast, aortic distensibility was not associated with CRF (36). Two studies investigated the associations between carotid distensibility and CRF; only Ried-Larsen et al. (37) reported a positive relationship (36, 37). Two of the three studies documented a positive association between carotid arterial compliance and CRF (21, 37, 48), while aortic AI@75 (the AI at a standardized heart rate of 75 beats per minute) was inversely associated with CRF (47).

Only one study investigated the relationship between muscle strength (push-up and curl-ups) and CRF; no associations with vascular function were identified when evaluating these parameters (21).

Several studies compared levels of CRF to specific stiffness parameters. Reed et al. (48) evaluated this relationship and found significantly lower compliance in large arteries in the first (up to 10 laps) and second quartile (21 to 32 laps) compared to the highest quartile (more than 43 laps) of CRF, recorded with a 20 m shuttle run; compliance of small arteries was lower in the second quartile of CRF compared to the fourth quartile.

Ried-Larsen et al. (37) divided CRF in four quartiles and observed significant lower carotid young's elastic modulus in the second to fourth quartiles compared to the first quartile; these results indicate stiffer arteries at lower fitness levels. Likewise, stiffness index was higher in the first quartile compared to the third and fourth quartile, and adolescents in CRF quartiles three and four showed significantly higher carotid compliance compared to adolescents within the first quartile. In a related study, very fit children and adolescents (>80th percentile) had lower elastic modulus, stiffness index and carotid PWV than low fit (<20th percentile) children and adolescents (21).

To summarize, most of the studies reported a beneficial association between vascular stiffness and CRF among children and adolescents; these findings are consistent with those reported in studies of, young, middle-aged and older adults (51–53). Nevertheless, it was difficult to compare studies to one another because of different methods used to measure both HRPF and vascular stiffness. Of interest, all studies, in which the 20 m shuttle run was used to assess HRPF, revealed a favorable association between stiffness parameters and HRPF.

## Exercise

Children and adolescents with obesity or hypertension typically have impaired arterial structure and function. Several groups have introduced intervention methods in an attempt to alter the vascular architecture among subjects in these groups. Besides, exercise also refers to children and adolescents who perform at a sustained level in organized sports club activities.

### Vascular Structure

Garcia-Hermoso et al. (54) published a comprehensive meta-analysis that included six studies that focused on the impact of aerobic, resistance or both aerobic and resistance exercises on vascular structure in obese populations aged between 6 and 18 years. Four of these studies (three aerobic, one aerobic, and resistance exercises) resulted in reductions in cIMT. Overall, the results indicate the changes in cIMT that result from exercise interventions were small to moderate (*g* = −0.306; 95% CI :−0.540 to −0.072, *p* = 0.011) with higher impact achieved in response to the longer interventions. Cayres et al. (41) also conclude that there are beneficial effects of exercise interventions on cIMT, but note that there are very few studies that examine interventions in healthy, non-obese populations.

In addition to these observations, there are several more recent interventional studies investigated that explore the impact of exercise on vascular structure (Table 4). For example, obese boys were subjected to a 12-week high-intensity intermittent training (HIIT) of 8 × 2 min at 90% peak power output or a supra-HIIT of 2 × 20 s at 170% peak power output; these strategies resulted in significant reductions in cIMT of 0.02 mm in both intervention groups, but had no impact on the diameter of the brachial artery (55).

**Table 4:** Overview of studies investigating the impact of exercise on vascular structure and function.

**References**	***N***	**Age**	**Intervention**	**Duration of intervention**	**Vascular measurement**	**Parameter of vascular structure**	**Parameter of vascular function**	**Results**	**Statistics**
**STUDIES IN PEDIATRIC POPULATIONS AT RISK**									
Chuensiri et al. (55)	48 obese boys (16 HIIT, 16 supra-HIIT, 16 controls)	8–12	HIIT (8 × 2 min at 90% peak power output)supra-HIIT (2 × 20 s at 170% peak power output)	3 times per week, 12 weeks	Ultrasound, automatic vascular screening device	cIMT brachial artery diameter	brachial PWV	↓brachial PWV in HIIT	*p* < 0.05
								↓brachial PWV supra-HIIT	*p* < 0.05
								mo time*group interaction in brachial PWV	-
								↓cIMT in HIIT	*p* < 0.05
								↓cIMT in supra-HIIT	*p* < 0.05
								mo time*group interaction in cIMT	-
								no change in brachial artery diameter	-
Horner et al. (22)	81 obese adolescents (30 aerobic exercise, 27 resistance exercise, 24 controls)	12–18	Aerobic exercise: 60 min moderate training on treadmill, elliptical or ergometer resistance exercise: 10 whole-body exercises (8–12 repetitions)	3 times per week, 3 months	Ultrasound, automatic device	cIMT	aortic PWV	no change in cIMT no change aortic PWV	- -
Seeger et al. (56)	7 DMI children	10.9 ± 1.5	30 min interval running and 10 min warm-up and cool-down	2 times per week, 18 weeks	Ultrasound	cIMT carotid artery diameter wall:lumen-ratio		no changes in parameters of arterial structure	-
Son et al. (57)	40 obese prehypertensive girls (20 intervention, 20 controls)	15 ± 1	60 min combined resistance and aerobic exercise	3 times per week, 12 weeks	Applanation tonometry		brachial PWV	↓brachial PWV in intervention group	*p* < 0.05
Sung et al. (23)	40 obese prehypertensive girls (20 intervention, 20 controls)	14–16	5 min warm-up, 40 min rope jumping variations, 5 min cool-down	5 times per week, 12 weeks	Applanation tonometry		brachial PWV	↓brachial PWV in intervention group	*p* ≤ 0.05
Wong et al. (58)	30 obese girls (15 intervention, 15 controls)	15.2 ± 1.2 15.3 ± 1.1	60 min combined resistance and aerobic exercise	3 times per week, 12 weeks	Applanation tonometry		brachial PWV	↓brachial PWV in intervention group	*p* < 0.05
**POPULATION-BASED STUDIES**									
Hacke et al. (59)	135 pre-schoolers (92 intervention group, 43 controls)	4.8 ± 0.8	45 min exercise lessons	2 times per week, 6 months	Oscillometric device		aortic PWV	no change in aortic PWV	-
Weston et al. (60)	101 adolescents (41 intervention, 60 controls)	14.0 ± 0.3	4 to 7 repetitions of 45 s drills (soccer, dance, boxing, basketball) and 90 s recovery	3 times per week, 10 weeks	Ultrasound	cIMT		no change in cIMT	-
**STUDIES IN YOUNG ATHLETES**									
Demirel et al. (61)	33 elite male wrestlers 35 matches controls	15.9 ± 0.9 16.0 ± 0.8			Ultrasound Applanation tonometry	cIMT	arterial compliance arterial distensibility diastolic wall stress elastic modulus	↓cIMT in athletes	*p* = 0.01
								↑diastolic wall stress in athletes	*p* = 0.03
								no difference in compliance	-
								no difference in distensibility	-
								no difference in elastic modulus	-

*HIIT, high-intensity intermittent training; DMI, diabetes mellitus type 1; cIMT, carotid intima-media thickness, PWV, pulse wave velocity; AI, aortic augmentation index*.

By contrast, seven children with type 1 diabetes mellitus were enrolled in an 18-week interval running intervention (30 min intervention, 10 min cool-down, two sessions per week); no significant changes in carotid artery diameter, cIMT or wall:lumen-ratio were detected (56).

To the best of our knowledge, there is only one published study that investigated the impact of an exercise intervention (high-intensity interval training) in a school-based population; this study also revealed no significant reduction in cIMT after 10 weeks (60). However, in young adolescent male wrestlers, cIMT measurements were significantly lower when compared to age- and sex-matched controls (61).

Taken together, the published results suggest that exercise interventions in groups of young participants may result in reduced thickening of the arterial walls, but the overall impact depends on duration and intensity of the intervention and the nature of the target groups. Given the current prevalence of overweight and obesity among children and adolescents, future population-based studies are needed to determine definitively whether exercise leads to an improved vascular health in these populations.

### Vascular Function

Exercise interventions on obese and/or prehypertensive children and adolescents have shown promising results toward the goal of reducing arterial stiffness. The study noted earlier in which 48 obese boys were challenged with a 12-week HIIT or supra-HIIT resulted in significant reductions in brachial PWV in both intervention groups (55). In another study, a combined resistance and aerobic exercise intervention (50 min, three times per week) in prehypertensive adolescent girls resulted in significant reductions in brachial-ankle PWV after 12 weeks (57). Similarly, a rope-jumping intervention (50 min, five times per week) resulted in reduced brachial-ankle PWVs in a study with obese prehypertensive girls (23). Likewise, Wong et al. (58) found that combined resistance and aerobic exercise (60 min, 3 times per week) in obese girls led to a significant reduction of brachial PWV compared to a control group after three months.

By contrast, Horner et al. (22) found no change in aortic PWV in obese adolescents who participated in aerobic (treadmill training, 60–75% of VO_2peak_) or resistance (whole-body exercises) interventions for a period of three months. Moreover, Hacke et al. (59) implemented preschool exercise lessons (45 min, two times per week for six months) but was unable to detect any improvement in aortic PWV in the intervention group compared to controls.

We identified only one study that evaluated vascular function in young athletes; this study reported no significant differences in arterial compliance, distensibility and elastic modulus among young adolescent male wrestlers vs. age- and sex-matched controls (61).

In summary, four out of five studies that we evaluated were conducted in children and adolescents with increased cardiovascular risk (obesity, hypertension), reported reductions in vascular stiffness after an exercise intervention. As such, we can conclude that exercise interventions with at least moderate levels of intensity can reduce vascular stiffness among pediatric populations at risk. Horner et al. (22) and Hacke et al. (59) reported no reductions in body mass index in response to these interventions; improvement in vascular function might be linked to a change in anthropometric parameters. It is also possible that the intensity of their intervention was too low to promote measurable changes in vascular function. The need to implement exercise programs in order to improve the health of an increasing number of inactive children and adolescents is supported globally. Further studies should take into account the intensity and duration of interventions to improve the design and therefore effectiveness of these programs.

## Conclusion

In conclusion, PA and HRPF are directly associated with improved vascular structure and function in children and adolescents as determined in population-based studies. Furthermore, interventions in pediatric populations at risk reveal promising results that suggest a role for moderate exercise in correcting early increases in vascular wall thickness and vascular stiffness.

The results of our study are comparable with the findings of adult studies. Here Thijssen et al. (62) showed in a comprehensive review that both physical activity and HRPF are inversely related to cIMT and that movement interventions lead to a significant reduction in wall thickness of the carotid, femoral and brachial arteries. The identical direction of the results was also observed in the correlation between activity, fitness and exercise and vascular function in adults (63–65).

It was difficult to perform critical comparisons among these studies given the variety of methods and measurements employed, especially in studies that measured vascular function. Elmenhorst et al. (66) compared carotid PWV measured by ultrasound and aortic PWV measured by an oscillometric device and found significant lower values of carotid PWV (4.01 ± 0.44 m/s) compared aortic PWV (4.67 ± 0.34, *p* < 0.001). In addition to physiological and hemodynamic differences between the carotid artery and the aorta, HRPF, PA and exercise may have a varied impact on the function along the arterial tree. Regarding IMT, all included studies that measured the vascular structure used an ultrasound device. However, both the type of ultrasound device and the analysis method used, e.g. B-Mode imaging or radiofrequency multiple M-line analysis, influence the magnitude of IMT and, therefore, limit the comparability of the included studies. Thus, only one technique should be performed in prospective studies (67). Following Skilton et al. (68), the choice of location of IMT measurement—at the carotid artery or the aorta—should be decided depending on the target group and the individual goal of each study.

Likewise, we are unable to comment on the role of exercise in young athletes given the limited numer of studies on this subject. As athletes are of special interest with respect to cardiovascular adaptation to exercise, this target population should be the subject of further investigation focused on the duration and intensity of exercise and its impact on vascular structure and function (69).

Future studies are also needed to address the question of wether IMT:diameter-ratio or wall:lumen-ratio and the role of this parameters determining vascular patency. At this time, most studies evaluate only IMT, a parameter that provides a quantitative assessment of wall thickness but does not take into account any changes in the vessel diameter or lumen in response to HRPF, PA and/or specific exercise interventions. Finally, our findings suggest that individual exercise participation should be evaluated quantitatively because intensity and duration likely influence the effectiveness of these interventions.

## Author Contributions

LB conceptualized the study, reviewed the literature, and drafted the manuscript. HW, RO-F, and TS conceptualized the study and provided important input for drafting and revising of the manuscript.

### Conflict of Interest

The authors declare that the research was conducted in the absence of any commercial or financial relationships that could be construed as a potential conflict of interest.

## Abbreviations

AI: augmentation index
AI@75: augmentation index at heart rate 75/min
aIMT: aortic intima-media thickness
cIMT: carotid intima-media thickness
CRF: cardiorespiratory fitness
CVD: cardiovascular disease
HIIT: high-intensity intermittent training
HRPF: health-related physical fitness
IMT: intima-media thickness
LPA: light physical activity
LTPA: leisure-time physical activity
LV: left ventricle
MET: metabolic equivalent
MPA: moderate physical activity
MVPA: moderate-to-vigorous physical activity
PA: physical activity
PWV: pulse wave velocity
SED: sedentary behavior
VPA: vigorous physical activity.
